# Web searching for systematic reviews: a case study of reporting standards in the UK Health Technology Assessment programme

**DOI:** 10.1186/s13104-015-1079-y

**Published:** 2015-04-16

**Authors:** Simon Briscoe

**Affiliations:** Evidence Synthesis & Modelling for Health Improvement (ESMI), University of Exeter Medical School, South Cloisters, St Luke’s Campus, Exeter, EX1 2LU UK

**Keywords:** Systematic reviews, Literature searching, Web searching, World Wide Web, Reporting standards

## Abstract

**Background:**

Identifying literature for a systematic review requires searching a variety of sources. The main sources are typically bibliographic databases. Web searching using search engines and websites may be used to identify grey literature. Searches should be reported in order to ensure transparency and reproducibility.

This study assesses the reporting of web searching for systematic reviews carried out by the National Institute for Health Research (NIHR) Health Technology Assessment (HTA) programme (UK). The study also makes recommendations about reporting web searching for systematic reviews in order to achieve a reasonable level of transparency and reproducibility.

**Methods:**

Systematic reviews were identified by searching the HTA database via the Centre for Reviews and Dissemination (CRD) website. Systematic reviews were included in the study if they made reference to searching the web using either search engines or websites. A data-extraction checklist was designed to record how web searching was reported. The checklist recorded whether a systematic review reported: the names of search engines or websites; the dates they were searched; the search terms; the results of the searches; and, in the case of websites, whether a URL was reported.

**Results:**

554 HTA reports published between January 2004 and December 2013 were identified. 300 of these reports are systematic reviews, of which 108 report web searching using either a search engine or a website. Overall, the systematic reviews assessed in the study exhibit a low standard of web search reporting. In the majority of cases, the only details reported are the names of websites (n = 54) or search engines (n = 33). A small minority (n = 6) exhibit the highest standard of web search reporting.

**Conclusions:**

Most web search reporting in systematic reviews carried out on the UK HTA programme is not detailed enough to ensure transparency and reproducibility. Transparency of reporting could be improved by adhering to a reporting standard such as the standard detailed in the CRD systematic reviews methods guidance. Reproducibility is harder to achieve due to the frequency of changes to websites and search engines.

**Electronic supplementary material:**

The online version of this article (doi:10.1186/s13104-015-1079-y) contains supplementary material, which is available to authorized users.

## Background

Web searching is considered to be a supplementary search method for a systematic review. The main search method usually consists of bibliographic database searching, which is used to retrieve journal articles and conference abstracts. Although there is research suggesting that bibliographic databases can be adequately replaced by the web search engine Google Scholar, [1] the results are contested by information professionals [2,3]. Instead, web searching is typically used for retrieving grey literature, [4] i.e. literature which is “produced on all levels of government, academics, business and industry in print and electronic formats, but which is not controlled by commercial publishers” (the so-called “Luxembourg definition”) [5].

Web searching for a systematic review should be reported to the extent that the search strategy and results are *transparent* [4]. This enables researchers to assess the quality of a search strategy. Transparent reporting also aims to ensure that the searches and results are *reproducible*, which allows researchers to repeat the search process to test or update the results. There are, however, variables which limit the reproducibility of web searching, such as changes to website content or URL addresses.

Although transparent and reproducible web search reporting can only be achieved imperfectly, the principles remain important in the context of a systematic review. This is clear from the widely cited PRISMA statement on reporting standards for systematic reviews, which states that “[t]he value of a systematic review depends on what was done, what was found, and the clarity of reporting.” [6].

### Current standards for web search reporting

At present, web search reporting standards for systematic reviews vary in detail. This is demonstrated by contrasting the guidance of three major organisations which produce methods guidance for systematic reviews in the UK: (1) The National Institute for Health and Care Excellence (NICE); (2) the Cochrane Collaboration; and (3) the Centre for Reviews and Dissemination (CRD).

1. The National Institute for Health and Care Excellence
  NICE produce systematic review methods guidance for writing UK health technology assessment (HTA) reports [7]. Currently, there is nothing on the methods of reporting web searching in this guidance. A draft of updated guidance, available for public consultation between 1 April 2014 and 30 June 2014, includes the requirement that “supplementary searching techniques [which include web-searching] should follow the same principles of transparency and reproducibility as other search methods” [8]. However, there is no specific guidance on how to apply these principles to web searching.
2. The Cochrane Collaboration
  The Cochrane Handbook contains more detail on web searching than the NICE guidance. It advises printing or saving electronic copies of information from websites in the event that a webpage is altered or removed. The handbook also states that the date a website is accessed should be recorded and included with the referencing [9].
3. The Centre for Reviews and Dissemination
  CRD’s guidance for undertaking systematic reviews is more detailed than either NICE or Cochrane guidance. It states that “[i]nternet searching should be carried out in as structured a way as possible and the procedure documented”. It also provides a checklist which advises reporting: “the website, the URL, the date searched, any specific sections searched and the search terms used” [4].

The variations in guidance outlined here imply that web search reporting is inconsistently carried out. This may compromise the transparency and reproducibility of the searching. As such, it is useful to assess the quality of web search reporting in systematic reviews and, if necessary, make recommendations for changes to practice. Scoping literature searches carried out in MEDLINE, Web of Science and Library, Information and Technology Abstracts did not reveal any studies that assess web search reporting for systematic reviews. (The MEDLINE scoping search is reproduced in Additional file 1). This study seeks to rectify this lack of research.

### Objectives

This study assesses the reporting of web searching for systematic reviews carried out on the UK HTA programme^a^. The UK HTA programme is commissioned and funded by the National Institute for Health Research (NIHR), and provides systematic reviews for NHS decision-making bodies such as NICE [10]. The study also makes recommendations about reporting web searching for systematic reviews in order to achieve a reasonable level of transparency and reproducibility. These recommendations are based on the existing systematic review guidance in the UK [4,7,9].

## Methods

### Search strategy

Systematic reviews were identified by searching the HTA database via the CRD website [11]. The phrase “NIHR Health Technology Assessment programme” (i.e. the standardised indexing term for NIHR (UK) HTA reports in the HTA database) was searched using the “Any field” search box. Searching was carried out in August 2013 and the results were date limited from 2004 to date (i.e. August 2013). The results were exported to Endnote X7 and the full text of each report was retrieved from the online NIHR Journals Library [12]. An update search was carried out in September 2014 and date limited to end of 2013. Duplicates from the first half of 2013 retrieved in the original search were deleted in Endnote. The resulting Endnote library contained UK HTA reports from 2004 to 2013.

### Selection criteria

Reports were included in the study if they were a systematic review and made reference to either searching the web using search engines, or searching the web by browsing websites. Search engines were defined as web interfaces which search the World Wide Web, including meta-search engines which search the World Wide Web via a combination of search engines. Examples of search engines include Google and AltaVista, and examples of meta-search engines include Dogpile and Ixquick. Websites were defined as web pages accessed via a common domain name that were not also search engines.

Subject gateways (for example, the grey literature database, OpenGrey) were excluded from the study because they often organise information using similar standards and tools to bibliographic databases. For example, they often use controlled vocabularies for indexing and offer advanced search interfaces with author, title, keyword and subject heading search options [13]. It was considered that these features meant that the required reporting standard would be different to other websites and search engines, more akin to the traditional methods used for bibliographic databases. Web searching using ongoing trials registries and theses catalogues were excluded for the same reason.

### Data collection

A data-extraction checklist was constructed to record how web searching was reported. The checklist was developed by manually inspecting the NICE, Cochrane and CRD guidelines for details of what should be reported. In addition, key word searching for the terms “web”, “internet”, “grey literature” and “supplementary” was carried out in online versions of the guidelines. (References to web searching in the guidelines are summarised in the background section of this study). A test screening of 25 reports was carried out by two independent reviewers to ensure the checklist was adequate for the task. The final checklist recorded whether a systematic review reported:

1. the names of search engines or websites;
2. the dates they were searched;
3. the search terms;
4. the results of the searches;

and in the case of websites,

5) whether a URL (i.e. web address) was recorded.

A URL was not deemed a necessary reporting detail for search engines as there is usually only a single UK web address which is the same as the name of the search engine.

## Results

554 HTA reports published between January 2004 and December 2013 were identified. 300 of these reports are systematic reviews, of which 108 report web searching using either a search engine or a website and meet the additional selection criteria (see the methods section for selection criteria). The breakdown of web searching methods used in the systematic reviews are as follows:

- 28 systematic reviews report the use of both a search engine and a website;
- 48 systematic reviews report web-searching using a search engine;
- 88 systematic reviews report web-searching using a website.

The 108 included studies are listed in Additional file 2. 38 of these studies were written on behalf of NICE.

### Web searching using search engines

**Figure 1:**
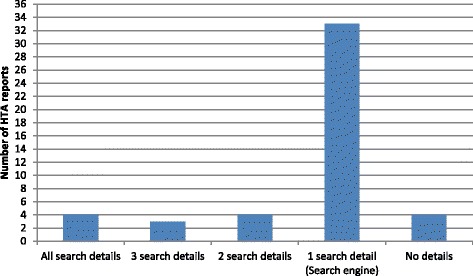
Details reported about web searching using search engines.

Figure 1 shows how frequently the details in the data-extraction checklist were reported in the 48 systematic reviews which report the use of a search engine: 33 report 1 detail, which in all cases was the name of the search engine used; 4 report everything in the checklist, i.e. search engine name, search terms, dates searched and the results; 3 report the search engine name and 2 additional details, e.g. name of search engine, search terms and dates searched; 4 report the search engine name and 1 additional detail. 4 systematic reviews report no details, i.e. they only state that an unnamed search engine was used as part of the search strategy.

Regarding the particular details that are reported about web searching using a search engine, the date searched (n = 7), search terms (n = 8) and results (n = 7) are reported almost equally. Google is the most frequently used search engine (n = 21) (see Table 1). This is closely followed by the meta-search engine Copernic (n = 17). Google Scholar (n = 9), AltaVista (n = 5) and Dogpile (n = 2) are less frequently reported.

**Table 1:** **Search engines cited by reports**

**Search engine**	**No. of reports**
Google	21
Copernic	17
Google Scholar	9
AltaVista	5
Dogpile	2

The most frequent reporting method was a short reference to the fact that a search engine was used. For example, “Keyword searching of the World Wide Web was undertaken using the Google search engine” [14]. By contrast, a systematic review by Rodgers *et al.*, which reports all the details in the data-extraction checklist in an appendix, detailed that Google was searched on the 1st and 2nd December 2003 resulting in eleven studies matching the inclusion criteria [15]. A list of search terms was also included in the appendix.

A systematic review by Carr *et al.*, which also reported all the details in the checklist, included a brief narrative of how the search engine results were screened, as follows: “The first 100 results returned by each search strategy were scanned for relevance and those judged to be potentially relevant were followed up” [16]. The review also documents the number of hits each search string returned, alongside a list of search terms.

### Web searching using websites

**Figure 2:**
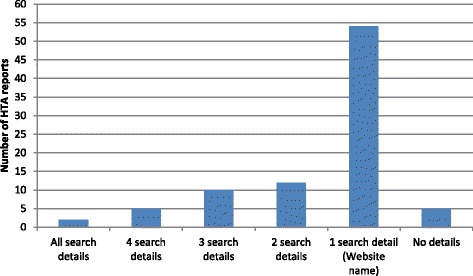
Details reported about web searching using websites (1).

Figure 2 shows how frequently the details in the data-extraction checklist were reported in the 88 systematic reviews which report the use of a website: 54 report 1 detail, which in all cases was the name of the website; 2 report everything, i.e. website name and URL, search terms, dates searched and the results; 5 report the website name and 3 additional details; 10 report the website name and 2 additional details; 12 report the website name and 1 additional detail. 5 systematic reviews report no details, only stating that unnamed websites were searched.

**Figure 3:**
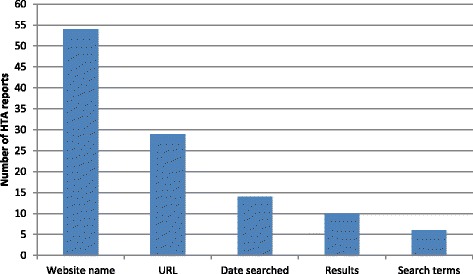
Details reported about web searching using websites (2).

Figure 3 shows how frequently each detail was reported, i.e. website name, URL, date searched, results and search terms. The most frequently reported detail is the name of the website (n = 54), followed by the URL (n = 29). The date searched (n = 14) and the results (n = 10) are less frequently reported, and the search terms (n = 6) are the least reported.

The websites searched included medical societies, UK, European and North American government websites, NGO and charity websites, manufacturer websites and National Economic Unit websites.

The most common reporting method is a list of the websites searched. A report by McKenna *et al.* is one of two systematic reviews which provide all the details in the checklist for website searching [17]. The appendix details that the US Food and Drug Administration (FDA) website was searched on the 13th December 2007, and supplies the URL. McKenna *et al.* also provides a brief narrative about how the search strategy was adapted for use on the FDA website. This is reproduced in the following excerpt:

The search interface to the FDA website is very simple and the search strategy had to be adapted accordingly.

Two searches were carried out. All of the FDA website was searched.

*Search 1*

(“all of the words”) EECP.

*Search 2*

(“with the exact phrase”) External counterpulsation (“without the words”) EECP [17].

The number of hits retrieved is reported as 97 [17].

## Discussion

There are a small number of systematic reviews in this study that exhibit a high standard of web search reporting. However, in the majority of cases, the only details reported are the names of websites or search engines. This limits the transparency and reproducibility of the search strategies. The remainder of this study will consider how best to achieve transparency and reproducibility when reporting web searching. It will also consider some aspects of the web which limit the reproducibility of web searching, even when the most transparent reporting standards are used.

### Transparency

In order to be transparent, a web search report should document the search strategy and the results. The reporting standard detailed in the CRD handbook cites most of the points needed to achieve transparency when reporting the use of *websites*, i.e. the report should list the website name, the URL, the date searched, any specific sections searched and the search terms used [4]. In addition, it may be useful to record the overall number of results retrieved, as this indicates what someone attempting to reproduce the search should expect to see. (If the number is radically different, it indicates that the website has been updated or altered). Any results which are included in the systematic review should certainly be reported, including in the bibliography.

The Cochrane handbook’s recommendation to print or save copies of the results is perhaps mainly useful for record keeping rather than for ensuring transparency: reproductions of webpages are unlikely to be included in the published version of a systematic review due to copyright or limited space.

Achieving transparency when reporting the use of *search engines* is somewhat different to websites, due to the relative size of the World Wide Web compared to a website. A website is usually divided into sections or relatively small, so that all of the results can be screened. By contrast, a search engine will often return hundreds of thousands of results which are impractical to screen. For example searching Google for the phrase “diabetes prevention strategy” retrieves 1,430,000 hits. Because the results are unlikely to be screened in full, the transparency of reporting is not improved by simply detailing the number of results. Instead, the focus should be on reporting how results were selected for screening. For example, the systematic review by Carr reported that only the first 100 results returned by a search engine were screened [16].

The recommended details to report for web searching using websites and search engines are as follows:

*Websites:*

- Name
- URL
- Dates searched
- Search terms (including any specific sections searched)
- Results

*Search Engines:*

- Name
- Dates searched
- Search terms
- How the results were selected

### Reproducibility

Reproducibility is measured by the ability to achieve the same results as the original search. In large part, reproducibility depends on the transparency of reporting. However, it also depends on eliminating unknown variables from the search strategy. For example, if the same search term retrieves different results on different days of the week, the reproducibility of a search is compromised, regardless of the transparency of reporting. In the context of searching bibliographic databases, eliminating unknown variables can almost be taken for granted: bibliographic databases are typically stable and return the same results on different dates and for different users. Unknown variables play a more significant role in web searching, making reproducibility difficult to achieve.

The reasons why these variables occur varies for websites and search engines. Regarding websites, their location, ownership, structure and contents may frequently change [13]. In the short term, there will be some stability. But in the time between completing a systematic review and updating it, perhaps several years later, the same URLs and search terms are likely to retrieve different results or result in broken web links.

Regarding search engines, the algorithms used to retrieve information may change over time and according to the user. Google, the most popular search engine in this study (and, also, worldwide [18]), has been the subject of detailed analysis of the way in which search results vary for the same search terms. Blakeman has written how Google may subject its users to twelve or more retrieval experiments every time they search [19]. For example, the search engine will sometimes offer subtly different results for the same search terms to different sets of users, with the aim of determining the most popular set of results. Similarly, Google records the search history of users by keeping a record of every internet device’s (e.g. computer) uniquely assigned internet protocol (IP) address. Using this information Google tries to tailor search results to what it thinks the user wants to see. Pariser has coined the term “filter bubble” to describe the personal bias this introduces to Google searches [20].

These issues severely curtail the reproducibility of results when web searching, even if the most transparent reporting standards are applied. Reproducibility is not impossible but it relies in part on luck and is unlikely to be achieved with consistency. It may be possible to eliminate some of the variables discussed here by using a search engine which does not try to tailor search results to user preferences. An example is DuckDuckGo, which does not record IP addresses [19]. Table 2 summarises the usefulness of transparent reporting in relation to the ability to reproduce search results.

**Table 2:** **The usefulness of transparent web search reporting in relation to the reproducibility of search results**

	**Websites**	**Search engines**
**Name**	Essential information for reproducing search.	Essential information for reproducing search.
**URL**	Useful information but URLs may change due to re-organisation of website.	n/a
**Dates searched**	Useful information but searching at a later date may retrieve entirely different results rather than updating the original results (see results, below).	Useful information but searching at a later date may retrieve entirely different results rather than update the original results (see results below).
**Search terms**	Essential information for reproducing search.	Essential information for reproducing search.
**Results**	Useful information but results may change due to changes to web page content or removal of online documents, such as PDFs or spreadsheets.	Useful information but results may change due to search engine algorithm changes or personalised results.

However, transparency is achievable, and remains a useful principle independently of reproducibility. Transparency allows a search strategy to be critiqued, allowing the reader to assess whether any useful information is likely to have been missed.

### Limitations of the study

Search strategies for HTA reports typically focus on retrieving high level evidence, such as randomized controlled trials and systematic reviews. The type of web searching most likely to usefully supplement a search for high level evidence will focus on grey literature generated by trial data. This data is usually indexed in ongoing trials registries or conference proceedings [21]. Web searching for this information was excluded from the study, because of the relative sophistication of searching trials registries and conference proceedings. As such, the web searching assessed was likely to have been a peripheral part of an already supplementary search method. The relative unimportance of this web search activity in relation to the outcome of a systematic review may have influenced the thoroughness with which it was reported.

## Conclusions

Most web search reporting in systematic reviews carried out on the UK HTA programme is not detailed enough to ensure even a limited level of transparency and reproducibility. Adherence to the recommendations outlined in this study (largely based on the CRD guidance) would improve the *transparency* of web search reporting. Due to unknown variables, the *reproducibility* of web searching is not reliably achieved even with the most detailed reporting standard. As such, web search reporting should aim for a *reasonable* level of transparency and reproducibility, rather than transparency and reproducibility *simpliciter*. Development of the CRD, NICE and Cochrane guidance to reflect this finding would be instructive for authors of systematic reviews.

## Endnote

^a^This study was first presented at the InterTASC Information Specialist Sub-Group workshop on the use of information in UK HTA reports, 9th July 2014, University of Exeter.

## Additional files

Additional file 1:
**MEDLINE scoping search.**
Additional file 2:
**Included studies.**
