# Erratum to: Web searching for systematic reviews: a case study of reporting standards in the UK Health Technology Assessment programme

**DOI:** 10.1186/s13104-016-1971-0

**Published:** 2016-03-15

**Authors:** Simon Briscoe

**Affiliations:** Evidence Synthesis & Modelling for Health Improvement (ESMI), University of Exeter Medical School, South Cloisters, St Luke’s Campus, Exeter, EX1 2LU UK

## Erratum to: BMC Research Notes (2015) 8:153 DOI 10.1186/s13104-015-1079-y

Unfortunately, the original version of this article [1] contained an error. In both the Abstract and the Results section the number of HTA reports containing the name of the website, (n = 54) should have read (n = 83).

This also affects the data in Figure 3 (Fig. 1 here), where “Website name” should also have read 83 on the chart rather than 54. A correct version of Figure 3 can be seen below.

**Fig. 1:**
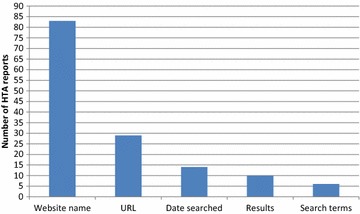
Details reported about web searching using websites (2)

## References

[1] Briscoe S (2015). Web searching for systematic reviews: a case study of reporting standards in the UK Health Technology Assessment programme. BMC Res Notes.

