# Supplementary material for: Web searching for systematic reviews: a case study of reporting standards in the UK Health Technology Assessment programme
Source: BMC Res Notes. 2015 Apr 16;8:153. doi: 10.1186/s13104-015-1079-y (PMC4406036; doi:10.1186/s13104-015-1079-y)
Supplement: Additional file 1: — MEDLINE scoping search. [file 13104_2015_1079_MOESM1_ESM.docx]

**Additional file 1: MEDLINE scoping search**

Database: MEDLINE

Host: Ovid

Date searched: 1/10/2014

Number of results: 2,867

Search strategy:

1. (search* adj3 (web* or "world wide web" or internet or online or "gr?y literature")).tw.
2. (googl* or "alta vista" or altavista or yahoo or dogpile or metacrawler or blog* or "social media").tw.
3. ("search engine*" or "metasearch engine*").tw.
4. or/1-3
5. Search Engine/
6. Information Seeking Behavior/
7. exp internet/
8. or/5-7
9. 4 or 8
10. ("systematic review*" or "meta-analys?s" or metaanalys?s).tw.
11. meta-analysis.pt.
12. ("health technology assessment*" or HTA).tw.
13. or/10-12
14. 9 and 13
15. limit 14 to (english language and yr="1990 -Current")
