# Supplementary material for: Web searching for systematic reviews: a case study of reporting standards in the UK Health Technology Assessment programme
Source: BMC Res Notes. 2015 Apr 16;8:153. doi: 10.1186/s13104-015-1079-y (PMC4406036; doi:10.1186/s13104-015-1079-y)
Supplement: Additional file 2: — Included studies. [file 13104_2015_1079_MOESM2_ESM.docx]

**Additional file 2: Included studies**

1. Abubakar I, Pimpin L, Ariti C, Beynon R, Mangtani P, Sterne JA, Fine PE, Smith PG, Lipman M, Elliman D *et al*: **Systematic review and meta-analysis of the current evidence on the duration of protection by bacillus Calmette-Guerin vaccination against tuberculosis**. *Health technology assessment (Winchester, England)* 2013, **17**(37):1-372, v-vi.

2. Adi Y, Juarez-Garcia A, Wang D, Jowett S, Frew E, Day E, Bayliss S, Roberts T, Burls A: **Oral naltrexone as a treatment for relapse prevention in formerly opioid-dependent drug users: a systematic review and economic evaluation**. *Health technology assessment (Winchester, England)* 2007, **11**(6):iii-iv, 1-85.

3. Albon E, Tsourapas A, Frew E, Davenport C, Oyebode F, Bayliss S, Arvanitis T, Meads C: **Structural neuroimaging in psychosis: a systematic review and economic evaluation**. *Health technology assessment (Winchester, England)* 2008, **12**(18):iii-iv, ix-163.

4. Ara R, Blake L, Gray L, Hernandez M, Crowther M, Dunkley A, Warren F, Jackson R, Rees A, Stevenson M *et al*: **What is the clinical effectiveness and cost-effectiveness of using drugs in treating obese patients in primary care? A systematic review**. *Health technology assessment (Winchester, England)* 2012, **16**(5):iii-xiv, 1-195.

5. Ara R, Tumur I, Pandor A, Duenas A, Williams R, Wilkinson A, Paisley S, Chilcott J: **Ezetimibe for the treatment of hypercholesterolaemia: a systematic review and economic evaluation**. *Health technology assessment (Winchester, England)* 2008, **12**(21):iii, xi-xiii, 1-212.

6. Ashfaq K, Yahaya I, Hyde C, Andronis L, Barton P, Bayliss S, Chen YF: **Clinical effectiveness and cost-effectiveness of stem cell transplantation in the management of acute leukaemia: a systematic review**. *Health technology assessment (Winchester, England)* 2010, **14**(54):iii-iv, ix-xi, 1-141.

7. Beynon R, Hawkins J, Laing R, Higgins N, Whiting P, Jameson C, Sterne JA, Vergara P, Hollingworth W: **The diagnostic utility and cost-effectiveness of selective nerve root blocks in patients considered for lumbar decompression surgery: a systematic review and economic model**. *Health technology assessment (Winchester, England)* 2013, **17**(19):1-88, v-vi.

8. Black C, Clar C, Henderson R, MacEachern C, McNamee P, Quayyum Z, Royle P, Thomas S: **The clinical effectiveness of glucosamine and chondroitin supplements in slowing or arresting progression of osteoarthritis of the knee: a systematic review and economic evaluation**. *Health technology assessment (Winchester, England)* 2009, **13**(52):1-148.

9. Black C, Sharma P, Scotland G, McCullough K, McGurn D, Robertson L, Fluck N, MacLeod A, McNamee P, Prescott G *et al*: **Early referral strategies for management of people with markers of renal disease: a systematic review of the evidence of clinical effectiveness, cost-effectiveness and economic analysis**. *Health technology assessment (Winchester, England)* 2010, **14**(21):1-184.

10. Bond M, Pavey T, Welch K, Cooper C, Garside R, Dean S, Hyde C: **Systematic review of the psychological consequences of false-positive screening mammograms**. *Health technology assessment (Winchester, England)* 2013, **17**(13):1-170, v-vi.

11. Bowling A, Rowe G, Lambert N, Waddington M, Mahtani KR, Kenten C, Howe A, Francis SA: **The measurement of patients' expectations for health care: a review and psychometric testing of a measure of patients' expectations**. *Health technology assessment (Winchester, England)* 2012, **16**(30):i-xii, 1-509.

12. Brazier J, Tumur I, Holmes M, Ferriter M, Parry G, Dent-Brown K, Paisley S: **Psychological therapies including dialectical behaviour therapy for borderline personality disorder: a systematic review and preliminary economic evaluation**. *Health technology assessment (Winchester, England)* 2006, **10**(35):iii, ix-xii, 1-117.

13. Bridle C, Palmer S, Bagnall AM, Darba J, Duffy S, Sculpher M, Riemsma R: **A rapid and systematic review and economic evaluation of the clinical and cost-effectiveness of newer drugs for treatment of mania associated with bipolar affective disorder**. *Health technology assessment (Winchester, England)* 2004, **8**(19):iii-iv, 1-187.

14. Brown T, Pilkington G, Bagust A, Boland A, Oyee J, Tudur-Smith C, Blundell M, Lai M, Martin Saborido C, Greenhalgh J *et al*: **Clinical effectiveness and cost-effectiveness of first-line chemotherapy for adult patients with locally advanced or metastatic non-small cell lung cancer: a systematic review and economic evaluation**. *Health technology assessment (Winchester, England)* 2013, **17**(31):1-278.

15. Brush J, Boyd K, Chappell F, Crawford F, Dozier M, Fenwick E, Glanville J, McIntosh H, Renehan A, Weller D *et al*: **The value of FDG positron emission tomography/computerised tomography (PET/CT) in pre-operative staging of colorectal cancer: a systematic review and economic evaluation**. *Health technology assessment (Winchester, England)* 2011, **15**(35):1-192, iii-iv.

16. Burch J, Epstein D, Baba-Akbari A, Weatherly H, Fox D, Golder S, Jayne D, Drummond M, Woolacott N: **Stapled haemorrhoidectomy (haemorrhoidopexy) for the treatment of haemorrhoids: a systematic review and economic evaluation**. *Health technology assessment (Winchester, England)* 2008, **12**(8):iii-iv, ix-x, 1-193.

17. Burch J, Paulden M, Conti S, Stock C, Corbett M, Welton NJ, Ades AE, Sutton A, Cooper N, Elliot AJ *et al*: **Antiviral drugs for the treatment of influenza: a systematic review and economic evaluation**. *Health technology assessment (Winchester, England)* 2009, **13**(58):1-265, iii-iv.

18. Burr JM, Botello-Pinzon P, Takwoingi Y, Hernandez R, Vazquez-Montes M, Elders A, Asaoka R, Banister K, van der Schoot J, Fraser C *et al*: **Surveillance for ocular hypertension: an evidence synthesis and economic evaluation**. *Health technology assessment (Winchester, England)* 2012, **16**(29):1-271, iii-iv.

19. Burr JM, Mowatt G, Hernandez R, Siddiqui MA, Cook J, Lourenco T, Ramsay C, Vale L, Fraser C, Azuara-Blanco A *et al*: **The clinical effectiveness and cost-effectiveness of screening for open angle glaucoma: a systematic review and economic evaluation**. *Health technology assessment (Winchester, England)* 2007, **11**(41):iii-iv, ix-x, 1-190.

20. Carlton J, Karnon J, Czoski-Murray C, Smith KJ, Marr J: **The clinical effectiveness and cost-effectiveness of screening programmes for amblyopia and strabismus in children up to the age of 4-5 years: a systematic review and economic evaluation**. *Health technology assessment (Winchester, England)* 2008, **12**(25):iii, xi-194.

21. Carr SM, Lhussier M, Forster N, Geddes L, Deane K, Pennington M, Visram S, White M, Michie S, Donaldson C *et al*: **An evidence synthesis of qualitative and quantitative research on component intervention techniques, effectiveness, cost-effectiveness, equity and acceptability of different versions of health-related lifestyle advisor role in improving health**. *Health technology assessment (Winchester, England)* 2011, **15**(9):iii-iv, 1-284.

22. Castelnuovo E, Stein K, Pitt M, Garside R, Payne E: **The effectiveness and cost-effectiveness of dual-chamber pacemakers compared with single-chamber pacemakers for bradycardia due to atrioventricular block or sick sinus syndrome: systematic review and economic evaluation**. *Health technology assessment (Winchester, England)* 2005, **9**(43):iii, xi-xiii, 1-246.

23. Chambers D, Paulden M, Paton F, Heirs M, Duffy S, Craig D, Hunter J, Wilson J, Sculpher M, Woolacott N: **Sugammadex for the reversal of muscle relaxation in general anaesthesia: a systematic review and economic assessment**. *Health technology assessment (Winchester, England)* 2010, **14**(39):1-211.

24. Chen YF, Jobanputra P, Barton P, Bryan S, Fry-Smith A, Harris G, Taylor RS: **Cyclooxygenase-2 selective non-steroidal anti-inflammatory drugs (etodolac, meloxicam, celecoxib, rofecoxib, etoricoxib, valdecoxib and lumiracoxib) for osteoarthritis and rheumatoid arthritis: a systematic review and economic evaluation**. *Health technology assessment (Winchester, England)* 2008, **12**(11):1-278, iii.

25. Chen YF, Jobanputra P, Barton P, Jowett S, Bryan S, Clark W, Fry-Smith A, Burls A: **A systematic review of the effectiveness of adalimumab, etanercept and infliximab for the treatment of rheumatoid arthritis in adults and an economic evaluation of their cost-effectiveness**. *Health technology assessment (Winchester, England)* 2006, **10**(42):iii-iv, xi-xiii, 1-229.

26. Clar C, Barnard K, Cummins E, Royle P, Waugh N: **Self-monitoring of blood glucose in type 2 diabetes: systematic review**. *Health technology assessment (Winchester, England)* 2010, **14**(12):1-140.

27. Clar C, Cummins E, McIntyre L, Thomas S, Lamb J, Bain L, Jobanputra P, Waugh N: **Clinical and cost-effectiveness of autologous chondrocyte implantation for cartilage defects in knee joints: systematic review and economic evaluation**. *Health technology assessment (Winchester, England)* 2005, **9**(47):iii-iv, ix-x, 1-82.

28. Clark W, Jobanputra P, Barton P, Burls A: **The clinical and cost-effectiveness of anakinra for the treatment of rheumatoid arthritis in adults: a systematic review and economic analysis**. *Health technology assessment (Winchester, England)* 2004, **8**(18):iii-iv, ix-x, 1-105.

29. Collins R, Fenwick E, Trowman R, Perard R, Norman G, Light K, Birtle A, Palmer S, Riemsma R: **A systematic review and economic model of the clinical effectiveness and cost-effectiveness of docetaxel in combination with prednisone or prednisolone for the treatment of hormone-refractory metastatic prostate cancer**. *Health technology assessment (Winchester, England)* 2007, **11**(2):iii-iv, xv-xviii, 1-179.

30. Connock M, Frew E, Evans BW, Bryan S, Cummins C, Fry-Smith A, Li Wan Po A, Sandercock J: **The clinical effectiveness and cost-effectiveness of newer drugs for children with epilepsy. A systematic review**. *Health technology assessment (Winchester, England)* 2006, **10**(7):iii, ix-118.

31. Connock M, Juarez-Garcia A, Jowett S, Frew E, Liu Z, Taylor RJ, Fry-Smith A, Day E, Lintzeris N, Roberts T *et al*: **Methadone and buprenorphine for the management of opioid dependence: a systematic review and economic evaluation**. *Health technology assessment (Winchester, England)* 2007, **11**(9):1-171, iii-iv.

32. Cranny G, Elliott R, Weatherly H, Chambers D, Hawkins N, Myers L, Sculpher M, Eastwood A: **A systematic review and economic model of switching from non-glycopeptide to glycopeptide antibiotic prophylaxis for surgery**. *Health technology assessment (Winchester, England)* 2008, **12**(1):iii-iv, xi-xii, 1-147.

33. Czoski-Murray C, Karnon J, Jones R, Smith K, Kinghorn G: **Cost-effectiveness of screening high-risk HIV-positive men who have sex with men (MSM) and HIV-positive women for anal cancer**. *Health technology assessment (Winchester, England)* 2010, **14**(53):iii-iv, ix-x, 1-101.

34. Czoski-Murray C, Lloyd Jones M, McCabe C, Claxton K, Oluboyede Y, Roberts J, Nicholl JP, Rees A, Reilly CS, Young D *et al*: **What is the value of routinely testing full blood count, electrolytes and urea, and pulmonary function tests before elective surgery in patients with no apparent clinical indication and in subgroups of patients with common comorbidities: a systematic review of the clinical and cost-effective literature**. *Health technology assessment (Winchester, England)* 2012, **16**(50):i-xvi, 1-159.

35. Czoski-Murray C, Warren E, Chilcott J, Beverley C, Psyllaki MA, Cowan J: **Clinical effectiveness and cost-effectiveness of pioglitazone and rosiglitazone in the treatment of type 2 diabetes: a systematic review and economic evaluation**. *Health technology assessment (Winchester, England)* 2004, **8**(13):iii, ix-x, 1-91.

36. de Verteuil R, Imamura M, Zhu S, Glazener C, Fraser C, Munro N, Hutchison J, Grant A, Coyle D, Coyle K *et al*: **A systematic review of the clinical effectiveness and cost-effectiveness and economic modelling of minimal incision total hip replacement approaches in the management of arthritic disease of the hip**. *Health technology assessment (Winchester, England)* 2008, **12**(26):iii-iv, ix-223.

37. Dretzke J, Cummins C, Sandercock J, Fry-Smith A, Barrett T, Burls A: **Autoantibody testing in children with newly diagnosed type 1 diabetes mellitus**. *Health technology assessment (Winchester, England)* 2004, **8**(22):iii-xi, 1-183.

38. Dretzke J, Frew E, Davenport C, Barlow J, Stewart-Brown S, Sandercock J, Bayliss S, Raftery J, Hyde C, Taylor R: **The effectiveness and cost-effectiveness of parent training/education programmes for the treatment of conduct disorder, including oppositional defiant disorder, in children**. *Health technology assessment (Winchester, England)* 2005, **9**(50):iii, ix-x, 1-233.

39. Dretzke J, Sandercock J, Bayliss S, Burls A: **Clinical effectiveness and cost-effectiveness of prehospital intravenous fluids in trauma patients**. *Health technology assessment (Winchester, England)* 2004, **8**(23):iii, 1-103.

40. Fayter D, Nixon J, Hartley S, Rithalia A, Butler G, Rudolf M, Glasziou P, Bland M, Stirk L, Westwood M: **A systematic review of the routine monitoring of growth in children of primary school age to identify growth-related conditions**. *Health technology assessment (Winchester, England)* 2007, **11**(22):iii, xi-xii, 1-163.

41. Fleeman N, McLeod C, Bagust A, Beale S, Boland A, Dundar Y, Jorgensen A, Payne K, Pirmohamed M, Pushpakom S *et al*: **The clinical effectiveness and cost-effectiveness of testing for cytochrome P450 polymorphisms in patients with schizophrenia treated with antipsychotics: a systematic review and economic evaluation**. *Health technology assessment (Winchester, England)* 2010, **14**(3):1-157, iii.

42. Fox M, Mealing S, Anderson R, Dean J, Stein K, Price A, Taylor RS: **The clinical effectiveness and cost-effectiveness of cardiac resynchronisation (biventricular pacing) for heart failure: systematic review and economic model**. *Health technology assessment (Winchester, England)* 2007, **11**(47):iii-iv, ix-248.

43. Garside R, Pitt M, Anderson R, Mealing S, Roome C, Snaith A, D'Souza R, Welch K, Stein K: **The effectiveness and cost-effectiveness of cinacalcet for secondary hyperparathyroidism in end-stage renal disease patients on dialysis: a systematic review and economic evaluation**. *Health technology assessment (Winchester, England)* 2007, **11**(18):iii, xi-xiii, 1-167.

44. Garside R, Pitt M, Anderson R, Rogers G, Dyer M, Mealing S, Somerville M, Price A, Stein K: **The effectiveness and cost-effectiveness of carmustine implants and temozolomide for the treatment of newly diagnosed high-grade glioma: a systematic review and economic evaluation**. *Health technology assessment (Winchester, England)* 2007, **11**(45):iii-iv, ix-221.

45. Garside R, Stein K, Castelnuovo E, Pitt M, Ashcroft D, Dimmock P, Payne L: **The effectiveness and cost-effectiveness of pimecrolimus and tacrolimus for atopic eczema: a systematic review and economic evaluation**. *Health technology assessment (Winchester, England)* 2005, **9**(29):iii, xi-xiii,1-230.

46. Goodacre S, Thokala P, Carroll C, Stevens JW, Leaviss J, Al Khalaf M, Collinson P, Morris F, Evans P, Wang J: **Systematic review, meta-analysis and economic modelling of diagnostic strategies for suspected acute coronary syndrome**. *Health technology assessment (Winchester, England)* 2013, **17**(1):v-vi, 1-188.

47. Greenhalgh J, Knight C, Hind D, Beverley C, Walters S: **Clinical and cost-effectiveness of electroconvulsive therapy for depressive illness, schizophrenia, catatonia and mania: systematic reviews and economic modelling studies**. *Health technology assessment (Winchester, England)* 2005, **9**(9):1-156, iii-iv.

48. Harris J, Felix L, Miners A, Murray E, Michie S, Ferguson E, Free C, Lock K, Landon J, Edwards P: **Adaptive e-learning to improve dietary behaviour: a systematic review and cost-effectiveness analysis**. *Health technology assessment (Winchester, England)* 2011, **15**(37):1-160.

49. Hartwell D, Jones J, Baxter L, Shepherd J: **Peginterferon alfa and ribavirin for chronic hepatitis C in patients eligible for shortened treatment, re-treatment or in HCV/HIV co-infection: a systematic review and economic evaluation**. *Health technology assessment (Winchester, England)* 2011, **15**(17):i-xii, 1-210.

50. Hill RA, Boland A, Dickson R, Dundar Y, Haycox A, McLeod C, Mujica Mota R, Walley T, Bagust A: **Drug-eluting stents: a systematic review and economic evaluation**. *Health technology assessment (Winchester, England)* 2007, **11**(46):iii, xi-221.

51. Hislop J, Quayyum Z, Elders A, Fraser C, Jenkinson D, Mowatt G, Sharma P, Vale L, Petty R: **Clinical effectiveness and cost-effectiveness of imatinib dose escalation for the treatment of unresectable and/or metastatic gastrointestinal stromal tumours that have progressed on treatment at a dose of 400 mg/day: a systematic review and economic evaluation**. *Health technology assessment (Winchester, England)* 2011, **15**(25):1-178.

52. Hislop J, Quayyum Z, Flett G, Boachie C, Fraser C, Mowatt G: **Systematic review of the clinical effectiveness and cost-effectiveness of rapid point-of-care tests for the detection of genital chlamydia infection in women and men**. *Health technology assessment (Winchester, England)* 2010, **14**(29):1-97, iii-iv.

53. Hoyle M, Crathorne L, Peters J, Jones-Hughes T, Cooper C, Napier M, Tappenden P, Hyde C: **The clinical effectiveness and cost-effectiveness of cetuximab (mono- or combination chemotherapy), bevacizumab (combination with non-oxaliplatin chemotherapy) and panitumumab (monotherapy) for the treatment of metastatic colorectal cancer after first-line chemotherapy (review of technology appraisal No.150 and part review of technology appraisal No. 118): a systematic review and economic model**. *Health technology assessment (Winchester, England)* 2013, **17**(14):1-237.

54. Imamura M, Abrams P, Bain C, Buckley B, Cardozo L, Cody J, Cook J, Eustice S, Glazener C, Grant A *et al*: **Systematic review and economic modelling of the effectiveness and cost-effectiveness of non-surgical treatments for women with stress urinary incontinence**. *Health technology assessment (Winchester, England)* 2010, **14**(40):1-188, iii-iv.

55. Kaltenthaler E, Brazier J, De Nigris E, Tumur I, Ferriter M, Beverley C, Parry G, Rooney G, Sutcliffe P: **Computerised cognitive behaviour therapy for depression and anxiety update: a systematic review and economic evaluation**. *Health technology assessment (Winchester, England)* 2006, **10**(33):iii, xi-xiv, 1-168.

56. Kaltenthaler E, Vergel YB, Chilcott J, Thomas S, Blakeborough T, Walters SJ, Bouchier H: **A systematic review and economic evaluation of magnetic resonance cholangiopancreatography compared with diagnostic endoscopic retrograde cholangiopancreatography**. *Health technology assessment (Winchester, England)* 2004, **8**(10):iii, 1-89.

57. Kanis JA, Stevenson M, McCloskey EV, Davis S, Lloyd-Jones M: **Glucocorticoid-induced osteoporosis: a systematic review and cost-utility analysis**. *Health technology assessment (Winchester, England)* 2007, **11**(7):iii-iv, ix-xi, 1-231.

58. Karnon J, Peters J, Platt J, Chilcott J, McGoogan E, Brewer N: **Liquid-based cytology in cervical screening: an updated rapid and systematic review and economic analysis**. *Health technology assessment (Winchester, England)* 2004, **8**(20):iii, 1-78.

59. King S, Griffin S, Hodges Z, Weatherly H, Asseburg C, Richardson G, Golder S, Taylor E, Drummond M, Riemsma R: **A systematic review and economic model of the effectiveness and cost-effectiveness of methylphenidate, dexamfetamine and atomoxetine for the treatment of attention deficit hyperactivity disorder in children and adolescents**. *Health technology assessment (Winchester, England)* 2006, **10**(23):iii-iv, xiii-146.

60. Knight C, Hind D, Brewer N, Abbott V: **Rituximab (MabThera) for aggressive non-Hodgkin's lymphoma: systematic review and economic evaluation**. *Health technology assessment (Winchester, England)* 2004, **8**(37):iii, ix-xi, 1-82.

61. Liu J, Davidson E, Bhopal R, White M, Johnson M, Netto G, Deverill M, Sheikh A: **Adapting health promotion interventions to meet the needs of ethnic minority groups: mixed-methods evidence synthesis**. *Health technology assessment (Winchester, England)* 2012, **16**(44):1-469.

62. Maund E, Craig D, Suekarran S, Neilson A, Wright K, Brealey S, Dennis L, Goodchild L, Hanchard N, Rangan A *et al*: **Management of frozen shoulder: a systematic review and cost-effectiveness analysis**. *Health technology assessment (Winchester, England)* 2012, **16**(11):1-264.

63. McDaid C, Hartley S, Bagnall AM, Ritchie G, Light K, Riemsma R: **Systematic review of effectiveness of different treatments for childhood retinoblastoma**. *Health technology assessment (Winchester, England)* 2005, **9**(48):iii, ix-x, 1-145.

64. McKenna C, McDaid C, Suekarran S, Hawkins N, Claxton K, Light K, Chester M, Cleland J, Woolacott N, Sculpher M: **Enhanced external counterpulsation for the treatment of stable angina and heart failure: a systematic review and economic analysis**. *Health technology assessment (Winchester, England)* 2009, **13**(24):iii-iv, ix-xi, 1-90.

65. McKenna C, Wade R, Faria R, Yang H, Stirk L, Gummerson N, Sculpher M, Woolacott N: **EOS 2D/3D X-ray imaging system: a systematic review and economic evaluation**. *Health technology assessment (Winchester, England)* 2012, **16**(14):1-188.

66. Meadows A, Kaambwa B, Novielli N, Huissoon A, Fry-Smith A, Meads C, Barton P, Dretzke J: **A systematic review and economic evaluation of subcutaneous and sublingual allergen immunotherapy in adults and children with seasonal allergic rhinitis**. *Health technology assessment (Winchester, England)* 2013, **17**(27):vi, xi-xiv, 1-322.

67. Meads C, Auguste P, Davenport C, Malysiak S, Sundar S, Kowalska M, Zapalska A, Guest P, Thangaratinam S, Martin-Hirsch P *et al*: **Positron emission tomography/computerised tomography imaging in detecting and managing recurrent cervical cancer: systematic review of evidence, elicitation of subjective probabilities and economic modelling**. *Health technology assessment (Winchester, England)* 2013, **17**(12):1-323.

68. Mowatt G, Houston G, Hernandez R, de Verteuil R, Fraser C, Cuthbertson B, Vale L: **Systematic review of the clinical effectiveness and cost-effectiveness of oesophageal Doppler monitoring in critically ill and high-risk surgical patients**. *Health technology assessment (Winchester, England)* 2009, **13**(7):iii-iv, ix-xii, 1-95.

69. Mowatt G, Scotland G, Boachie C, Cruickshank M, Ford JA, Fraser C, Kurban L, Lam TB, Padhani AR, Royle J *et al*: **The diagnostic accuracy and cost-effectiveness of magnetic resonance spectroscopy and enhanced magnetic resonance imaging techniques in aiding the localisation of prostate abnormalities for biopsy: a systematic review and economic evaluation**. *Health technology assessment (Winchester, England)* 2013, **17**(20):vii-xix, 1-281.

70. Nelson EA, O'Meara S, Craig D, Iglesias C, Golder S, Dalton J, Claxton K, Bell-Syer SE, Jude E, Dowson C *et al*: **A series of systematic reviews to inform a decision analysis for sampling and treating infected diabetic foot ulcers**. *Health technology assessment (Winchester, England)* 2006, **10**(12):iii-iv, ix-x, 1-221.

71. Oliver S, Bagnall AM, Thomas J, Shepherd J, Sowden A, White I, Dinnes J, Rees R, Colquitt J, Oliver K *et al*: **Randomised controlled trials for policy interventions: a review of reviews and meta-regression**. *Health technology assessment (Winchester, England)* 2010, **14**(16):1-165, iii.

72. Orlando R, Pennant M, Rooney S, Khogali S, Bayliss S, Hassan A, Moore D, Barton P: **Cost-effectiveness of transcatheter aortic valve implantation (TAVI) for aortic stenosis in patients who are high risk or contraindicated for surgery: a model-based economic evaluation**. *Health technology assessment (Winchester, England)* 2013, **17**(33):1-86.

73. Pandor A, Eastham J, Beverley C, Chilcott J, Paisley S: **Clinical effectiveness and cost-effectiveness of neonatal screening for inborn errors of metabolism using tandem mass spectrometry: a systematic review**. *Health technology assessment (Winchester, England)* 2004, **8**(12):iii, 1-121.

74. Pandor A, Eggington S, Paisley S, Tappenden P, Sutcliffe P: **The clinical and cost-effectiveness of oxaliplatin and capecitabine for the adjuvant treatment of colon cancer: systematic review and economic evaluation**. *Health technology assessment (Winchester, England)* 2006, **10**(41):iii-iv, xi-xiv, 1-185.

75. Pandor A, Goodacre S, Harnan S, Holmes M, Pickering A, Fitzgerald P, Rees A, Stevenson M: **Diagnostic management strategies for adults and children with minor head injury: a systematic review and an economic evaluation**. *Health technology assessment (Winchester, England)* 2011, **15**(27):1-202.

76. Picot J, Hartwell D, Harris P, Mendes D, Clegg AJ, Takeda A: **The effectiveness of interventions to treat severe acute malnutrition in young children: a systematic review**. *Health technology assessment (Winchester, England)* 2012, **16**(19):1-316.

77. Ramsay C, Pickard R, Robertson C, Close A, Vale L, Armstrong N, Barocas DA, Eden CG, Fraser C, Gurung T *et al*: **Systematic review and economic modelling of the relative clinical benefit and cost-effectiveness of laparoscopic surgery and robotic surgery for removal of the prostate in men with localised prostate cancer**. *Health technology assessment (Winchester, England)* 2012, **16**(41):1-313.

78. Riemsma R, Al M, Corro Ramos I, Deshpande SN, Armstrong N, Lee YC, Ryder S, Noake C, Krol M, Oppe M *et al*: **SeHCAT [tauroselcholic (selenium-75) acid] for the investigation of bile acid malabsorption and measurement of bile acid pool loss: a systematic review and cost-effectiveness analysis**. *Health technology assessment (Winchester, England)* 2013, **17**(61):1-236.

79. Rithalia A, McDaid C, Suekarran S, Norman G, Myers L, Sowden A: **A systematic review of presumed consent systems for deceased organ donation**. *Health technology assessment (Winchester, England)* 2009, **13**(26):iii, ix-xi, 1-95.

80. Robertson C, Arcot Ragupathy SK, Boachie C, Dixon JM, Fraser C, Hernandez R, Heys S, Jack W, Kerr GR, Lawrence G *et al*: **The clinical effectiveness and cost-effectiveness of different surveillance mammography regimens after the treatment for primary breast cancer: systematic reviews registry database analyses and economic evaluation**. *Health technology assessment (Winchester, England)* 2011, **15**(34):v-vi, 1-322.

81. Robinson L, Hutchings D, Corner L, Beyer F, Dickinson H, Vanoli A, Finch T, Hughes J, Ballard C, May C *et al*: **A systematic literature review of the effectiveness of non-pharmacological interventions to prevent wandering in dementia and evaluation of the ethical implications and acceptability of their use**. *Health technology assessment (Winchester, England)* 2006, **10**(26):iii, ix-108.

82. Rodgers M, Epstein D, Bojke L, Yang H, Craig D, Fonseca T, Myers L, Bruce I, Chalmers R, Bujkiewicz S *et al*: **Etanercept, infliximab and adalimumab for the treatment of psoriatic arthritis: a systematic review and economic evaluation**. *Health technology assessment (Winchester, England)* 2011, **15**(10):i-xxi, 1-329.

83. Rodgers M, Hodges R, Hawkins J, Hollingworth W, Duffy S, McKibbin M, Mansfield M, Harbord R, Sterne J, Glasziou P *et al*: **Colour vision testing for diabetic retinopathy: a systematic review of diagnostic accuracy and economic evaluation**. *Health technology assessment (Winchester, England)* 2009, **13**(60):1-160.

84. Rodgers M, Nixon J, Hempel S, Aho T, Kelly J, Neal D, Duffy S, Ritchie G, Kleijnen J, Westwood M: **Diagnostic tests and algorithms used in the investigation of haematuria: systematic reviews and economic evaluation**. *Health technology assessment (Winchester, England)* 2006, **10**(18):iii-iv, xi-259.

85. Ross JR, Saunders Y, Edmonds PM, Patel S, Wonderling D, Normand C, Broadley K: **A systematic review of the role of bisphosphonates in metastatic disease**. *Health technology assessment (Winchester, England)* 2004, **8**(4):1-176.

86. Shepherd J, Jones J, Hartwell D, Davidson P, Price A, Waugh N: **Interferon alpha (pegylated and non-pegylated) and ribavirin for the treatment of mild chronic hepatitis C: a systematic review and economic evaluation**. *Health technology assessment (Winchester, England)* 2007, **11**(11):1-205, iii.

87. Shepherd J, Jones J, Takeda A, Davidson P, Price A: **Adefovir dipivoxil and pegylated interferon alfa-2a for the treatment of chronic hepatitis B: a systematic review and economic evaluation**. *Health technology assessment (Winchester, England)* 2006, **10**(28):iii-iv, xi-xiv, 1-183.

88. Shepherd J, Kavanagh J, Picot J, Cooper K, Harden A, Barnett-Page E, Jones J, Clegg A, Hartwell D, Frampton GK *et al*: **The effectiveness and cost-effectiveness of behavioural interventions for the prevention of sexually transmitted infections in young people aged 13-19: a systematic review and economic evaluation**. *Health technology assessment (Winchester, England)* 2010, **14**(7):1-206, iii-iv.

89. Simpson EL, Duenas A, Holmes MW, Papaioannou D, Chilcott J: **Spinal cord stimulation for chronic pain of neuropathic or ischaemic origin: systematic review and economic evaluation**. *Health technology assessment (Winchester, England)* 2009, **13**(17):iii, ix-x, 1-154.

90. Soares-Weiser K, Bravo Vergel Y, Beynon S, Dunn G, Barbieri M, Duffy S, Geddes J, Gilbody S, Palmer S, Woolacott N: **A systematic review and economic model of the clinical effectiveness and cost-effectiveness of interventions for preventing relapse in people with bipolar disorder**. *Health technology assessment (Winchester, England)* 2007, **11**(39):iii-iv, ix-206.

91. Stevenson M, Davis S, Lloyd-Jones M, Beverley C: **The clinical effectiveness and cost-effectiveness of strontium ranelate for the prevention of osteoporotic fragility fractures in postmenopausal women**. *Health technology assessment (Winchester, England)* 2007, **11**(4):1-134.

92. Stevenson M, Jones ML, De Nigris E, Brewer N, Davis S, Oakley J: **A systematic review and economic evaluation of alendronate, etidronate, risedronate, raloxifene and teriparatide for the prevention and treatment of postmenopausal osteoporosis**. *Health technology assessment (Winchester, England)* 2005, **9**(22):1-160.

93. Stevenson M, Lloyd-Jones M, Papaioannou D: **Vitamin K to prevent fractures in older women: systematic review and economic evaluation**. *Health technology assessment (Winchester, England)* 2009, **13**(45):iii-xi, 1-134.

94. Sutcliffe P, Connock M, Pulikottil-Jacob R, Kandala NB, Suri G, Gurung T, Grove A, Shyangdan D, Briscoe S, Maheswaran H *et al*: **Clinical effectiveness and cost-effectiveness of second- and third-generation left ventricular assist devices as either bridge to transplant or alternative to transplant for adults eligible for heart transplantation: systematic review and cost-effectiveness model**. *Health technology assessment (Winchester, England)* 2013, **17**(53):1-499, v-vi.

95. Sutcliffe P, Connock M, Shyangdan D, Court R, Kandala NB, Clarke A: **A systematic review of evidence on malignant spinal metastases: natural history and technologies for identifying patients at high risk of vertebral fracture and spinal cord compression**. *Health technology assessment (Winchester, England)* 2013, **17**(42):1-274.

96. Tappenden P, Campbell F, Rawdin A, Wong R, Kalita N: **The clinical effectiveness and cost-effectiveness of home-based, nurse-led health promotion for older people: a systematic review**. *Health technology assessment (Winchester, England)* 2012, **16**(20):1-72.

97. Tappenden P, Harnan S, Uttley L, Mildred M, Carroll C, Cantrell A: **Colistimethate sodium powder and tobramycin powder for inhalation for the treatment of chronic Pseudomonas aeruginosa lung infection in cystic fibrosis: systematic review and economic model**. *Health technology assessment (Winchester, England)* 2013, **17**(56):v-xvii, 1-181.

98. Thangaratinam S, Rogozinska E, Jolly K, Glinkowski S, Duda W, Borowiack E, Roseboom T, Tomlinson J, Walczak J, Kunz R *et al*: **Interventions to reduce or prevent obesity in pregnant women: a systematic review**. *Health technology assessment (Winchester, England)* 2012, **16**(31):iii-iv, 1-191.

99. Wang D, Cummins C, Bayliss S, Sandercock J, Burls A: **Immunoprophylaxis against respiratory syncytial virus (RSV) with palivizumab in children: a systematic review and economic evaluation**. *Health technology assessment (Winchester, England)* 2008, **12**(36):iii, ix-x, 1-86.

100. Warren E, Weatherley-Jones E, Chilcott J, Beverley C: **Systematic review and economic evaluation of a long-acting insulin analogue, insulin glargine**. *Health technology assessment (Winchester, England)* 2004, **8**(45):iii, 1-57.

101. Waugh N, Royle P, Craigie I, Ho V, Pandit L, Ewings P, Adler A, Helms P, Sheldon C: **Screening for cystic fibrosis-related diabetes: a systematic review**. *Health technology assessment (Winchester, England)* 2012, **16**(24):iii-iv, 1-179.

102. Whiting P, Gupta R, Burch J, Mota RE, Wright K, Marson A, Weishmann U, Haycox A, Kleijnen J, Forbes C: **A systematic review of the effectiveness and cost-effectiveness of neuroimaging assessments used to visualise the seizure focus in people with refractory epilepsy being considered for surgery**. *Health technology assessment (Winchester, England)* 2006, **10**(4):1-250, iii-iv.

103. Whiting P, Westwood M, Bojke L, Palmer S, Richardson G, Cooper J, Watt I, Glanville J, Sculpher M, Kleijnen J: **Clinical effectiveness and cost-effectiveness of tests for the diagnosis and investigation of urinary tract infection in children: a systematic review and economic model**. *Health technology assessment (Winchester, England)* 2006, **10**(36):iii-iv, xi-xiii, 1-154.

104. Willis BH, Barton P, Pearmain P, Bryan S, Hyde C: **Cervical screening programmes: can automation help? Evidence from systematic reviews, an economic analysis and a simulation modelling exercise applied to the UK**. *Health technology assessment (Winchester, England)* 2005, **9**(13):1-207, iii.

105. Wilson J, Connock M, Song F, Yao G, Fry-Smith A, Raftery J, Peake D: **Imatinib for the treatment of patients with unresectable and/or metastatic gastrointestinal stromal tumours: systematic review and economic evaluation**. *Health technology assessment (Winchester, England)* 2005, **9**(25):1-142.

106. Woloshynowych M, Rogers S, Taylor-Adams S, Vincent C: **The investigation and analysis of critical incidents and adverse events in healthcare**. *Health technology assessment (Winchester, England)* 2005, **9**(19):1-143, iii.

107. Woodroffe R, Yao GL, Meads C, Bayliss S, Ready A, Raftery J, Taylor RS: **Clinical and cost-effectiveness of newer immunosuppressive regimens in renal transplantation: a systematic review and modelling study**. *Health technology assessment (Winchester, England)* 2005, **9**(21):1-179, iii-iv.

108. Yao G, Albon E, Adi Y, Milford D, Bayliss S, Ready A, Raftery J, Taylor RS: **A systematic review and economic model of the clinical and cost-effectiveness of immunosuppressive therapy for renal transplantation in children**. *Health technology assessment (Winchester, England)* 2006, **10**(49):iii-iv, ix-xi, 1-157.
